# Nutrient criteria for surface waters under the European Water Framework Directive: Current state-of-the-art, challenges and future outlook

**DOI:** 10.1016/j.scitotenv.2019.133888

**Published:** 2019-12-10

**Authors:** Sandra Poikane, Martyn G. Kelly, Fuensanta Salas Herrero, Jo-Anne Pitt, Helen P. Jarvie, Ulrich Claussen, Wera Leujak, Anne Lyche Solheim, Heliana Teixeira, Geoff Phillips

**Affiliations:** a European Commission, Joint Research Centre (JRC), I-21027 Ispra, Italy; b Bowburn Consultancy, 11 Monteigne Drive, Bowburn, Durham DH6 5QB, UK; c Department of Geography, Nottingham University, Nottingham NG7 2RD, UK; d Environment Agency, Horizon House, Bristol BS1 5AH, UK; e Centre for Ecology and Hydrology, Wallingford, Oxfordshire OX10 8BB, UK; f Federal Environment Agency, Wörlitzer Platz 1, 06844 Dessau-Rosslau, Germany; g Norwegian Institute for Water Research (NIVA), Gaustadalleen 21, 0348 Oslo, Norway; h Department of Biology & CESAM, University of Aveiro, Campus de Santiago, 3810-193 Aveiro, Portugal; i School of Biological and Environmental Sciences, University of Stirling, Stirling FK9 4LA, UK

**Keywords:** Eutrophication, Ecological status, Phosphorus, Nitrogen, Inland waters, Coastal waters

## Abstract

The aim of European water policy is to achieve good ecological status in all rivers, lakes, coastal and transitional waters by 2027. Currently, more than half of water bodies are in a degraded condition and nutrient enrichment is one of the main culprits. Therefore, there is a pressing need to establish reliable and comparable nutrient criteria that are consistent with good ecological status.

This paper highlights the wide range of nutrient criteria currently in use by Member States of the European Union to support good ecological status and goes on to suggest that inappropriate criteria may be hindering the achievement of good status. Along with a comprehensive overview of nutrient criteria, we provide a critical analysis of the threshold concentrations and approaches by which these are set. We identify four essential issues: (1) Different nutrients (nitrogen and/or phosphorus) are used for different water categories in different countries. (2) The use of different nutrient fractions (total, dissolved inorganic) and statistical summary metrics (e.g., mean, percentiles, seasonal, annual) currently hampers comparability between countries, particularly for rivers, transitional and coastal waters. (3) Wide ranges in nutrient threshold values within shared water body types, in some cases showing more than a 10-fold difference in concentrations. (4) Different approaches used to set threshold nutrient concentrations to define the boundary between “good” and “moderate” ecological status. Expert judgement-based methods resulted in significantly higher (less stringent) good-moderate threshold values compared with data-driven approaches, highlighting the importance of consistent and rigorous approaches to criteria setting.

We suggest that further development of nutrient criteria should be based on relationships between ecological status and nutrient concentrations, taking into account the need for comparability between different water categories, water body types within these categories, and countries.

## Highlights

• We review the nutrient criteria used in Europe under the Water Framework Directive.
• Different nutrients (nitrogen and/or phosphorus) used in different waters and countries
• Different metrics used severely hamper comparability between countries.
• A wide range in nutrient criteria values are found even within common types.
• Approach to criteria setting has a significant effect on the nutrient criteria.

## Graphical abstract

**Figure:**
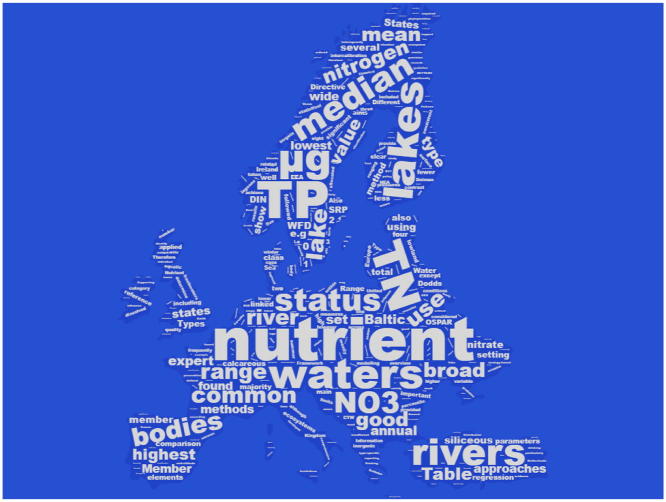

## 1 Introduction

European water policy aims to attain good ecological status (defined as no more than a slight deviation from near-natural conditions) in all rivers, lakes, coastal and transitional waters by 2015 or, at the latest, by 2027 (EC, 2000). However, by the most recent estimate (EEA, 2018; updated with recent data), 57% of rivers, 44% of lakes, 40% of coastal waters and 66% of transitional waters failed to achieve this. Various human drivers - agriculture, urbanization, hydropower generation and climate change – are responsible for this degradation of aquatic ecosystems (Borgwardt et al., 2019) with 50% of European water bodies impacted by more than one pressure and only 18% of surface water bodies with no significant pressures identified (EEA, 2018). Nutrient enrichment from both diffuse and point-sources remains one of the main reasons for the degradation of European water bodies (EEA, 2018; Grizzetti et al., 2017). Eutrophication threatens the provision of essential ecosystem services such as the supply of drinking water, recreation, and habitat provision for fish and wildlife (Culhane et al., 2019).

In Europe, the Water Framework Directive (WFD; EC, 2000) was adopted to protect and enhance the status of aquatic ecosystems. Under the WFD, ecological status is assessed in an integrated way through the use of biological quality elements (phytoplankton, benthic flora, benthic invertebrate and fish fauna) together with supporting hydromorphology and physico-chemical parameters, including nutrient conditions. The WFD stipulates that, at good ecological status, nutrient concentrations must “not exceed the levels established so as to ensure the functioning of the ecosystem and the achievement of values specified (for good status) for the biological quality elements” (Annex V, 1.2). Thus, the WFD does not provide nutrient concentration targets specifically but requires EU countries to determine type-specific nutrient criteria ensuring/supporting good ecological status. While a huge effort has been devoted to the development of the assessment methods using biological quality elements (BQEs) (Birk et al., 2012; Poikane et al., 2015), much less attention has been paid until recently to setting nutrient criteria (but see Dolman et al., 2016; Poikane et al., 2019; Salas Herrero et al., 2019). The requirement to intercalibrate the biological elements has led to a common view of good status (Birk et al., 2013; Kelly et al., 2014; Poikane et al., 2014); however, this process was not required for the supporting elements.

Recently, the focus has shifted from the assessment of ecological status towards identifying the management measures to reach good status (Carvalho et al., 2019). This has been fueled by the observation that, fifteen years after the WFD was introduced, less than half of all surface waters are in good ecological status, and there has been little or no improvement between 2009 and 2015 (EEA, 2018). Many reasons lie behind this lack of restoration success, including the lag-time between the implementation of management measures and ecosystem response (Jarvie et al., 2013; Sharpley et al., 2013). However, concerns about the weak linkages between management targets for nutrients and ecological status have also been raised (Carvalho et al., 2019), and the wide range of nutrient criteria established by different countries (Laane et al., 2005; Phillips and Pitt, 2016) raises the possibility that some of these may not be fit-for-purpose. Selecting appropriate nutrient criteria is vital to enable management of eutrophication of surface waters to achieve good ecological status. It is also important to ensure that management targets are consistent between countries and water categories, especially for transboundary water management (Hering et al., 2010; Dave and Munawar, 2014). Despite the critical importance of this question, to date no analysis nor overview has been conducted of the nutrient criteria used by member states to support good ecological status under the Water Framework Directive.

This study aims (i) to provide an overview of nutrient criteria, including thresholds for good ecological status and approaches used to set these; (ii) to identify whether nutrient criteria currently in use actually support good ecological status and are consistent between countries with similar water bodies; and, (iii) to provide recommendations for further work.

## 2 Material and methods

### 2.1 Collection of data on nutrient criteria and supporting information

Information on nutrient (phosphorus, P, and nitrogen, N; see Table 1 for a list of abbreviations and terminology) criteria were gathered from member states using a questionnaire. This included information on the nutrient parameters measured, units and metrics used and high-good and good-moderate class threshold concentrations for all types of surface water body as well as descriptions of the approaches used to derive nutrient criteria. Twenty-eight member states reported nutrient criteria for rivers, 26 for lakes, 23 for coastal waters and 18 for transitional waters (Table 2). A more detailed overview is presented as Supporting information for lakes and rivers (Table S1) and coastal and transitional waters (Table S2).

**Table 1:** Abbreviations and terminology used throughout this paper.

Abbreviation	Meaning
BQE	Biological quality element (biological communities, e.g., phytoplankton, benthic invertebrates, used to assess ecological status)
Classification	The WFD classification scheme for ecological status specifies five status classes: high, good, moderate, poor and bad, based on the extent of deviation from reference (=near-natural) conditions
DIN	Dissolved inorganic nitrogen: nitrate-N + nitrite-N + ammonium-N, measured on a filtered water sample
Ecological status	Assessment of the quality of the structure and functioning of surface water ecosystems; determined by biological quality elements, supported by hydromorphological and physico-chemical quality elements
Good ecological status	WFD objective for all water bodies; defined as a slight variation from undisturbed conditions
N	Nitrogen
Nutrient criteria	Water quality standards used to protect the waters from nutrient enrichment, consisting of nutrient parameter, metrics and threshold
• Parameter	N or P fraction measured (TN, TP, SRP etc.)
• Metrics	Statistics used (mean, median, percentile, annual, seasonal etc.)
• Threshold	Nutrient concentration representing the threshold between two quality classes; good-moderate class threshold – between good and moderate class
P	Phosphorus
SRP	Soluble Reactive Phosphorus: measures dissolved inorganic P + readily-hydrolysed (labile organic-, condensed- and colloidal-) P fractions in a filtered water sample with no digestion step^a^
TN	Total Nitrogen: measures dissolved + particulate inorganic and organic N fractions in an unfiltered water sample with a digestion step
TP	Total Phosphorus; measures TRP + dissolved and particulate organic P fractions in an unfiltered water sample with a digestion step.
TRP	Total Reactive Phosphorus: measures SRP + readily-hydrolysed particulate P fractions in an unfiltered water sample without a digestion step^a^
Water Framework Directive WFD	Water Framework Directive 2000/60/EC establishing a framework for community action in the field of water policy.

a See Jarvie et al. (2002).

**Table 2:** Number of countries reporting different nutrient criteria for rivers, lakes, coastal and transitional waters. x– nutrient criteria reported. Some countries report criteria for more than one region (e.g., France - Mediterranean Sea Region and France - North East Atlantic Sea region). BALT = Baltic Sea; MED = Mediterranean; NEA = North-East Atlantic.

Member state	Lakes		Rivers		Coastal waters			Transitional waters	
	P	N	P	N		P	N	P	N
Austria	x	Not used	x	x		No coastal and transitional waters			
Belgium-Flanders	x	x	x	x		x	x	x	x
Belgium-Wallonia	No lakes	No lakes	x	x		No coastal and transitional waters			
Bulgaria	x	x	x	x		x	x	x	x
Croatia	x	x	x	x		x	x	x	x
Cyprus	x	Not used	x	x		x	x	TRW not defined	
Czech Republic	x	Not used	x	x		No coastal and transitional waters			
Denmark	x	x	Not used			Not used		TRW not defined	
Estonia	x	x	x	x		x	x	TRW not defined	
Finland	x	x	x	x		x	x	TRW not defined	
France	x	x	x	x	MED	In development		x	x
					NEA	Not used	x	Not used	x
Germany	x	Not used	x	Not used	BALT	x	x	TRW not defined	
					NEA	x	x	x	x
Greece	x	x	x	x		x	x	x	x
Hungary	x	x	x	x		No coastal and transitional waters			
Ireland	x	Not used	x	x		Not used	x	x	Not used
Italy	x	Not used	x	x		x	Not used	x	x
Latvia	x	x	x	x		x	x	x	x
Lithuania	x	x	x	x		x	x	x	x
Luxemburg	No lakes	No lakes	x	x		No coastal and transitional waters			
Malta		In development							
Netherlands	x	x	x	x		Not used	x	Not used	x
Norway	x	x	x	x		x	x	TRW not defined	
Poland	x	x	x	x		x	x	x	x
Portugal	x	x	x	x		x	x	x	x
Romania	x	x	x	x		x	x	x	x
Slovakia	No lakes	No lakes	x	x		No coastal and transitional waters			
Slovenia	x	Not used	x	x		x	x	TRW not defined	
Spain	x	Not used	x	x	MED	x	x	x	x
					NEA	x	x	x	x
Sweden	x	Not used	x	Not used		x	x	x	x
United Kingdom	x	Not used	x	Not used		Not used	x	Not used	x
**Total**	**26**	**16**	**28**	**25**		**20**	**23**	**16**	**18**

### 2.2 Comparison of nutrient criteria within common types

When making comparisons of threshold concentrations between countries it is important to use similar water body types. The WFD leaves countries to determine their own water body typology, based on WFD Annex II type descriptors (System A or B), which has resulted in the description of >1500 national types of water bodies across Europe (Lyche Solheim et al., 2015, Lyche Solheim et al., 2019), too many for effective comparison. To simplify this those freshwater types that shared a similar descriptors (underlying geology, altitude, catchment size for rivers, surface area and mean depth for lakes) were grouped into European ‘broad types’ (Lyche Solheim et al., 2015, Lyche Solheim et al., 2019) (Tables S3 and S4).

In the case of coastal and transitional waters (CTW), reported national types were linked to regional ‘common types’ established for the intercalibration of the biological assessment systems (Tables S5 and S6).

For rivers, nutrient criteria for 680 national types were reported and 80% of records were matched to 20 broad types; for lakes, nutrient criteria for 369 national types were reported, 73% of which could be matched to broad types, while for coastal and transitional waters 231 national types were reported, of which 60% were allocated to common types.

Comparisons of nutrient criteria within broad types were carried out for those N and P parameters assessed by the majority of countries: total phosphorus (TP) and total nitrogen (TN) for rivers and lakes, and TP and dissolved inorganic nitrogen (DIN) for coastal and transitional waters. Most countries use mean (or median) values for nutrient criteria; however several countries use a 90th percentile summary metric. For comparison, the values of these percentiles were halved (analyses of a large UK data set for both TP and TRP suggested that a 90th percentile would be approximately double the value of a mean (Phillips and Pitt, 2016).

### 2.3 Methods to set the good-moderate threshold concentration

Each country was asked to summarise information about the way that the good-moderate threshold concentration was set. For both freshwaters and saline waters a wide range of methods were used; for simplification these have been grouped into six main approaches:

1. Regression between nutrient and biological response (Phillips et al., 2018);
2. Modelling – e.g. two countries predict reference TP in lakes from models of alkalinity and depth;
3. Distribution of nutrient concentrations in water bodies classified (using ecological criteria) as high, good and moderate status (Phillips et al., 2018);
4. Distribution of nutrient concentrations in all water bodies – using this approach the nutrient criteria are defined from an arbitrary percentile of the distribution of nutrient concentrations from all water bodies (Dodds and Welch, 2000).
5. Expert judgement, including values taken from the literature or from older European Directives. For example, for nitrate, the common use of the value 5.65 mg-N L^−1^ in freshwaters is likely to be derived from the guideline value of 25 mg L^−1^ of nitrate in the Nitrates Directive (91/676/EEC) or now repealed Drinking Water Directive (80/778/EC).
6. For coastal and transitional water, the so-called OSPAR Comprehensive Procedure is used widely. In this, a water body is considered to be an ‘Eutrophication Problem Area’ if actual status deviates 50% or more from reference conditions (OSPAR, 2013).

### 2.4 Statistical analysis

Statistical analysis of reported threshold values was performed using R 3.5.3. (R Core Team, 2019). The significance of different criteria setting methods was tested by Kruskal-Wallis Rank Sum Test and post-hoc Dunn's Test (Dinno, 2015). Effects were considered statistically significant at p < 0.05.

## 3 Results

### 3.1 Nutrient criteria: parameters and metrics

#### 3.1.1 Nutrient parameters and metrics in lakes and rivers

**Fig. 1:**
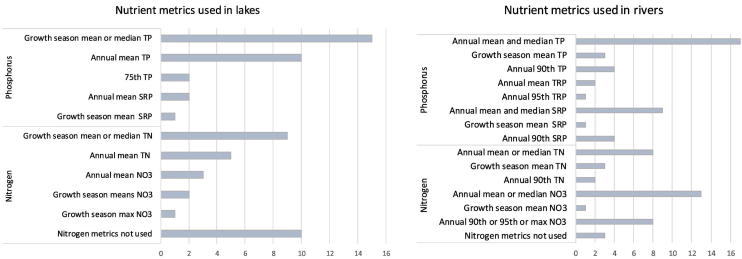
Metrics used to specify lake and river nutrient (phosphorus and nitrogen) good-moderate class criteria for ecological classification under the European Water Framework Directive. Further information about the breakdown in nutrient metrics used by individual member states is provided in the Supporting information, Table S1.

For lakes, all countries use TP and three additionally report soluble reactive phosphorus (SRP) (Fig. 1, Table 3). In contrast, only sixteen countries have a threshold value for N in lakes. The majority use TN (singly or in combination with nitrate), with two using only nitrate. Ten countries (38% of reporting countries) do not use N in lake assessment. Almost all countries use measures of central tendency (mean, median or geometric mean), with just Spain using an upper (75th) percentile.

**Table 3:** Nutrient parameters used by member states (number of countries and percentage of countries). CW–coastal waters, TW – transitional waters (estuaries, coastal lagoons etc.)

Nutrient parameters used in ecological classification	# of countries				% of all countries reported			
	Lakes^a^	Rivers^b^	CW^c^	TRW^c^	Lakes	Rivers	CW	TRW
Phosphorus parameters								
Total phosphorus (TP)	26	24	12	10	100%	82.8%	46.2%	52.6%
Total reactive phosphorus (TRP)	0	2	0	0	0	6.9%	0	0
Soluble reactive phosphorus (SRP)	3	14	14	12	11.5%	48.3%	53.9%	63.2%
Not using P parameters	0	1	5	3	0	3.4%	19.2%	15.8%
Nitrogen parameters								
Total nitrogen (TN)	14	13	10	8	53.9%	44.8%	38.5%	42.1%
Dissolved inorganic nitrogen (DIN)	0	0	9	11	0	0	32.1%	57.9%
Nitrate (NO_3_)	6	20	10	7	23.1%	69.0%	38.5%	36.8%
Not using N metrics	10	4	1	1	38.5%	13.8%	3.9%	5.3%
	26	29	23	19				

a Countries with no lakes (Belgium-Wallonia, Luxembourg, Slovakia) and Malta not included.

b Belgium-Flanders and Belgium-Wallonia counted separately, Malta not included.

c Countries may report criteria for more than one region (e.g., France - Mediterranean Sea Region and France - North East Atlantic Sea region).

For rivers, the majority (24 countries out of 29) reported TP thresholds, singly or in combination with SRP. However, four only reported soluble reactive phosphorus (SRP) (Austria and Spain) or TRP (Ireland and United Kingdom). Twenty-two countries reported threshold values for N in rivers, most use nitrate-N (20 countries) rather than TN (13 countries) while four countries do not use N metrics in rivers (Table 3). More countries used upper percentiles (e.g. 90th percentiles) summary metrics rather than measures of central tendency for rivers than was the case for lakes. One country (Denmark) has no nutrient thresholds for rivers for any nutrient parameter.

#### 3.1.2 Nutrient parameters in coastal and transitional waters

**Fig. 2:**
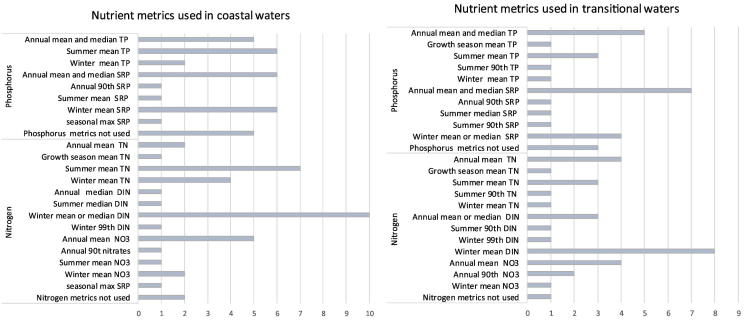
Metrics used to specify coastal and transitional waters nutrient (phosphorus and nitrogen) good-moderate class criteria for ecological classification under the European Water Framework Directive. Further information about the breakdown in nutrient metrics used by individual member states is provided in the Supporting information, Table S2.

In coastal waters, P is used for assessments by all countries in the Baltic, Mediterranean and the Black Sea, while four countries in the North East Atlantic use only N (France, Ireland, Netherlands, and United Kingdom) (Fig. 2, Table S2). The most frequently used parameters and metrics are summer mean TP, annual mean/median SRP and winter mean SRP (all used by six countries). Only Italy and Denmark do not use N but a wide variety of N parameters are in use (in total, 18 different metrics) with mean or median winter DIN (eight countries) and mean summer TN being the most frequently used parameter (seven countries).

P is used by almost all countries for assessments of transitional waters (Fig. 2, Table S2) apart from a few in the North East Atlantic (France, Netherlands, and United Kingdom). The most widely-used parameters and metrics are annual mean or median SRP (seven countries) and annual mean or median TP (five countries). In transitional waters, N is also used by all countries, except Ireland. The most frequently used parameters are winter mean DIN (used by eight countries), followed by annual mean, summer mean TN and annual mean nitrate (all used by four countries).

### 3.2 Comparison of nutrient thresholds by water body type

#### 3.2.1 Lake phosphorus and nitrogen thresholds

**Fig. 3:**
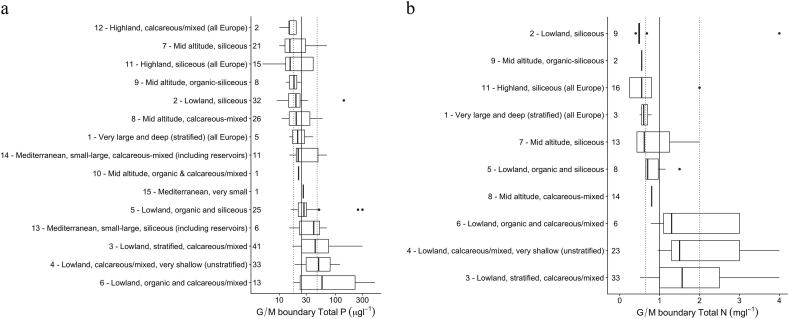
Range of reported good/moderate lake total phosphorus (a) and total nitrogen (b) threshold values grouped by broad types. Numbers show the number of national types allocated to each broad type. Types ordered by median value of reported thresholds, dotted lines show interquartile range for all broad types.

Across Europe, good-moderate TP threshold concentrations in lakes vary from 5 to 500 μg TP L^−1^ (median 27.5 μg TP L^−1^; interquartile range 20–50 μg TP L^−1^). The lowest values were found in highland lakes, siliceous lakes, as well as large deep lakes and mid-altitude lakes, with most of the thresholds being <40 μg TP L^−1^ (Fig. 3). In contrast, the highest thresholds, mostly >40 μg TP L^−1^, were reported in the lowland calcareous and Mediterranean lake types.

For several broad lake types, a wide range of TP values was reported for the good/moderate threshold concentrations. The highest range was recorded for Type 6 (lowland, calcareous and organic lakes) (18–500 μg TP L^−1^) and Type 3 (lowland, calcareous or mixed, stratified lakes) (18 to 300 μg TP L^−1^). This may reflect genuine natural differences in sensitivity or background nutrient concentrations of different lake types not captured within the broad type or different views of the TP concentrations required to support good status. The lake type with the highest boundary values is type 4, which is the lowland, calcareous, very shallow, unstratified lakes (from 20 to 300 μg TP L^−1^).

TN good-moderate thresholds range from 0.25 to 4.0 mg TN L^−1^ (median 1.0 mg TN L^−1^; interquartile range 0.7–2.5 mg TN L^−1^) (Fig. 3). In contrast to P, most countries have fewer type-specific thresholds, with eight countries having three or fewer different values covering all of their lake types. The lowest values reported were found in the siliceous lake types, typically with good/moderate thresholds below 1.0 mg TN L^−1^ (Fig. 3). The highest values were reported for the calcareous lake types, mostly between 1.0 and 3.0 mg TN L^−1^.

#### 3.2.2 River phosphorus and nitrogen thresholds

**Fig. 4:**
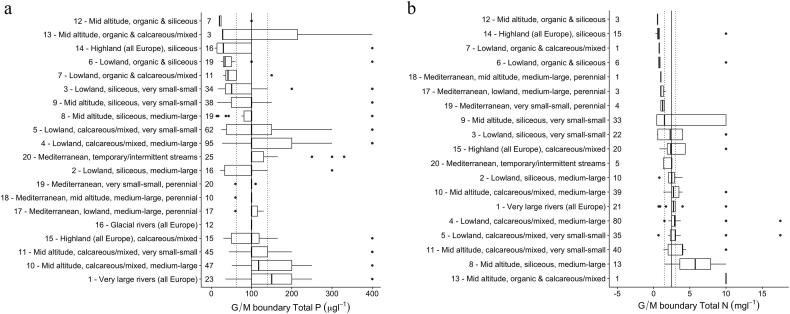
Range of reported good/moderate river total phosphorus (a) and total nitrogen (b) threshold values grouped by broad types. Numbers show the number of national types allocated to each broad type. Types ordered by median value of reported boundary, dotted lines show interquartile range for all broad types.

The reported TP threshold concentrations in rivers, in addition to being higher than those reported for lakes, were more variable across Europe, ranging from 8 to 660 μg TP L^−1^ (Fig. 4). The median threshold was 100 μg TP L^−1^, a commonly-used value particularly for lowland calcareous rivers (Types 3, 4, 5). In general there was a less clear gradation of threshold concentrations across the broad types than for lakes, although the lowest range of threshold concentrations were found in some of the siliceous river types (median ≤ 50 μg TP L^−1^) and highest in calcareous river types (median ≥ 100 μg TP L^−1^).

In comparison to lakes, the majority of countries reported fewer P boundary concentrations, despite having as many or more types for rivers as for lakes. Nine countries have only a single (national) threshold which is applied to all river types, while five countries have just two threshold concentrations, despite having many river types.

There is a wide range of good-moderate TN threshold concentrations (Fig. 4), ranging from 0.25 mg TN L^−1^ to 35 mg TN L^−1^ (median 2.5 mg TN L^−1^). As for lakes, there are far fewer national N threshold concentrations than there are national types, with the same concentration often applied to several river types. The lowest TN threshold concentrations (for types with >4 countries) are found in Types 14 (the highland siliceous rivers) and 6 (lowland organic and siliceous), with higher values in the calcareous river types. However, there is more variation than for lakes, with a less obvious gradation from upland siliceous to lowland calcareous river types. Several types have outliers (annual mean 10 mg TN L^−1^ and annual 90th percentile 35 mg TN L^−1^) and Type 9 (mid-altitude siliceous) has a very wide range of values, from 0.43 to 10.0 mg TN L^−1^.

The range of nitrate-N boundary concentrations (not shown) was much more variable and there is little indication of any clear relationship with the broad types. There is also a clear influence of the relatively widespread use of criteria values of 5.6 mg N L^−1^ and 11.3 mg N L^−1^ by several countries, probably attributable to the guideline value for drinking water (25 mg NO_3_ L^−1^) from the now repealed Drinking Water Directive 80/778/EC and from the guideline value of 50 mg NO_3_ L^−1^ in the Nitrates Directive 91/676/EEC.

#### 3.2.3 Phosphorus and nitrogen thresholds in coastal and transitional waters

**Fig. 5:**
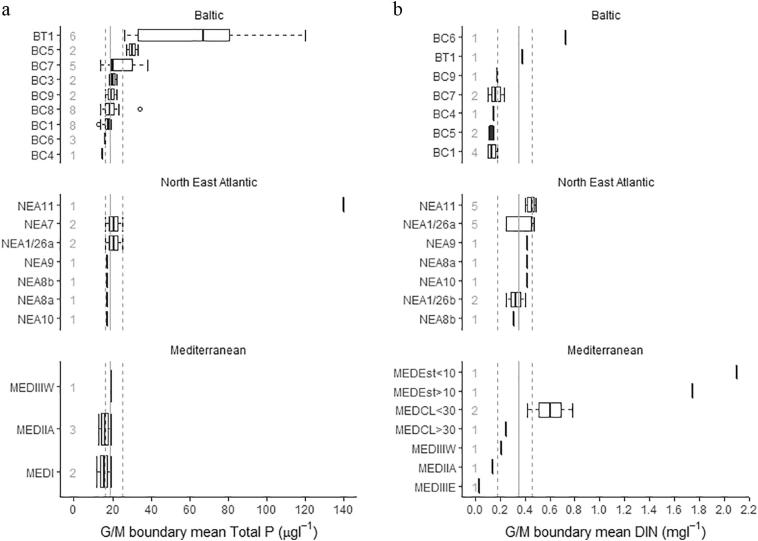
Range of reported good/moderate threshold values in coastal and transitional waters for total phosphorus (a) and dissolved inorganic nitrogen (b) criteria grouped by common types. Numbers show the number of national types allocated to each common type. Types ordered by marine region and median value of reported boundary, dotted lines show interquartile range for all common types, and solid line the median. Description of common types in supplementary material Table S2.

TP threshold concentrations in coastal waters range from 9.3 to 44 μg TP L^−1^; however most values are <25 μg TP L^−1^ (Fig. 5). The lowest concentrations were reported from the Mediterranean types (12–19 μg TP L^−1^) and from Baltic coastal type BC1 (13–19 μg TP L^−1^), while the highest values were found in the Baltic type B5 (27–33 μg TP L^−1^) and the most variable in the Baltic type BC7 (14–38 μg TP L^−1^). For type descriptions see Table S5.

For DIN, the lowest concentrations were reported from the Mediterranean type III E (0.03 N mg N L^−1^), an area of oligotrophic water and several Baltic types, BC1 (0.10–0.18 mg N L^−1^), BC5 (0.11–0.15 mg N L^−1^). Four countries sharing common type NEA 1/26a show concentrations in the range 0.25–0.47 mg N L^−1^).

For transitional waters, only a few countries have reported their threshold concentrations. The lowest concentrations are from Mediterranean estuaries (19–28 μg TP L^−1^) and the highest in North East Atlantic transitional water type NEA11-140 μg TP L^−1^. Baltic coastal lagoons BT1 show the greatest heterogeneity of reported threshold concentrations, ranging from 26 to 120 μg TP L^−1^, with differences probably related to varying influence of freshwaters in the lagoons.

The countries included in the transitional waters common type NEA 11 show very similar DIN threshold concentrations (0.42–0.49 mg N L^−1^) but this is not the case for Mediterranean coastal lagoons, shared by Greece and Italy (0.25–0.78 mg N L^−1^).

### 3.3 Methods to set good-moderate class threshold concentration

**Fig. 6:**
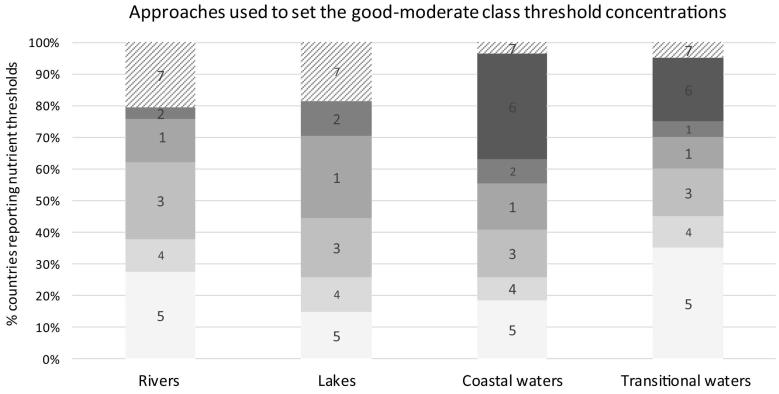
Approaches used to set nutrient threshold values for different water categories. 1 - regression between nutrient and biological response, 2 - modelling, 3 - distribution of nutrient concentrations in water bodies classified (using ecological criteria); 4 - distribution of nutrient concentrations in all water bodies using an arbitrary percentile, 5 - expert judgement, 6 - OSPAR (2013) approach, 7 – insufficient information. For details, see Section 2.3.

For lakes, the most common approach for establishing thresholds is the use of regression models, where nutrient concentration is related to a BQE, or part of a BQE (such as chlorophyll *a* concentration). This approach is less common for rivers, where the most commonly stated method is “expert judgement”. The distribution of nutrient concentrations in water bodies assigned a WFD status was the second most common approach for both lakes and rivers (Fig. 6).

For coastal waters, the most widely-used approach was the OSPAR approach where nutrient thresholds are presumed to deviate at maximum 50% from background concentrations (OSPAR, 2013), followed by expert judgement and pressure-response relationships between biological quality elements (mainly phytoplankton) and nutrients. For transitional waters, expert judgement is the most widely-used approach, followed by the OSPAR approach and distribution of classified water bodies (Fig. 6). In summary, countries used a wide variety of methods to establish threshold concentrations and in about half the cases the process is not linked to ecological status or an objective evaluation of reference conditions (i.e. approaches 4 and 5 in Section 2.3).

### 3.4 Comparison of nutrient thresholds by method of derivation

**Fig. 7:**
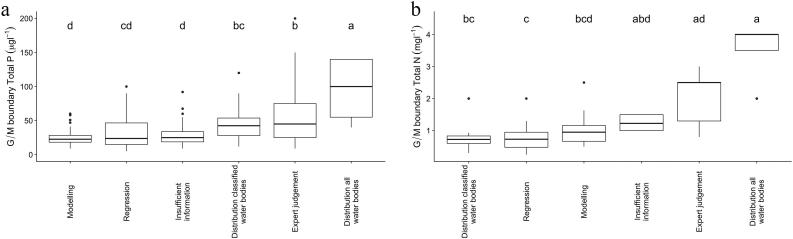
Range of good/moderate lake phosphorus (a) and nitrogen (b) threshold values grouped by method used to determine the value. Different letters indicate groups that are statistically different (p ≤ 0.05).

Differences in threshold concentrations were apparent when grouped by the method used to establish the criteria. For lake P, significantly higher (p < 0.001) threshold concentrations are found when the distribution of nutrient concentrations in all water bodies was used (median 100 μg TP L^−1^), followed by expert judgement (median 75 μg TP L^−1^) (Fig. 7). In contrast, approaches using modelling (median 22.6 μg TP L^−1^) and regression (23.8 μg TP L^−1^) tend to have lower threshold concentrations (although note that the results for modelling are only taken from three countries). The same applies for lake N, with the highest threshold concentrations reported when the distribution of nutrient concentrations in all water bodies (median 4 mg TN L^−1^) or expert judgement (median 2.5 mg TN L^−1^) are used, and the lowest when regression techniques or classified water bodies (median 0.73 mg TN L^−1^) are used.

**Fig. 8:**
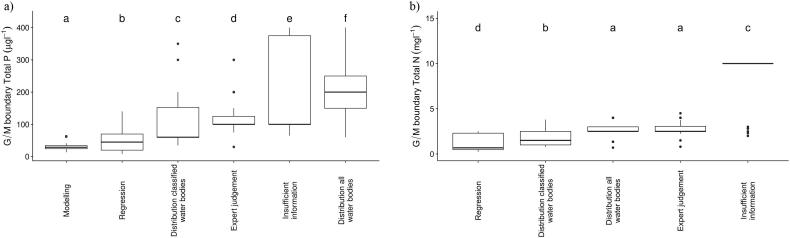
Range of good/moderate river phosphorus (a) and nitrogen (b) threshold values grouped method used to determine the value. Different letters indicate groups that are statistically different (p ≤ 0.05).

For river TP (Fig. 8), the highest threshold concentrations (median 200 μg TP L^−1^) are found when the distribution of nutrient concentrations in all water bodies is used, followed by expert judgement (median 100 μg TP L^−1^). Significantly lower (p < 0.001) threshold concentrations are obtained when the distribution of nutrient concentrations in classified water bodies (60 μg TP L^−1^) or regression (median 45 μg TP L^−1^) are used.

Again, for river N, the highest threshold concentrations are found when either expert judgement or the distribution of N concentration in all water bodies (for both median 2.5 mg TN L^−1^) is used. Significantly lower (p < 0.001) thresholds were obtained when the ecology was considered: regression method (median 0.68 mg TN L^−1^) and distribution of classified water bodies (median 1.5 mg TN L^−1^). No information was provided on the approaches used to set the highest TN threshold concentrations (mean annual values 10 mg TN L^−1^) (Fig. 8).

It was not possible to carry out this analysis for coastal and transitional waters due to the high heterogeneity of parameters and metrics used reported.

## 4 Discussion

### 4.1 Choice of nutrient: nitrogen, phosphorus or both?

There is a widespread belief, arising from ideas developed several decades ago, that P limits primary production in freshwaters (e.g. Hecky and Kilham, 1988; Vollenweider, 1976). This is reflected in the choice of nutrient criteria, as 10 countries do not use N for lakes and five countries do not use N for rivers (Germany, Ireland, Slovakia, Sweden, and UK).

However, the assertion that P alone limits primary production in lakes and that reducing P is sufficient to curb eutrophication (e.g. Schindler et al., 2008) has been challenged with evidence that N can play an important role in nutrient limitation of primary production in lakes (Scott and McCarthy, 2010; Dolman et al., 2016; Paerl et al., 2018). There is also extensive evidence, from bioassays and correlation analysis, that both P and N can limit primary production in rivers (e.g. Dodds and Welch, 2000; Francoeur, 2001; Dodds and Smith, 2016; Jarvie et al., 2018). For these reasons, both N and P should be considered when attempting to restore good ecological status in rivers too (Dodds et al., 2002; Dodds and Smith, 2016).

In coastal ecosystems, N is generally believed to limit primary production (Howarth and Marino, 2006; Tyrrell, 1999), leading to the widespread use of N, rather than P for assessing the status of these ecosystems. However, the situation differs among countries and regional seas. Baltic countries, for example, tend to use both N and P metrics, based on an understanding of eutrophication in this region (HELCOM, 2015). Generally, N limits phytoplankton growth in the coastal waters of the Baltic Sea, apart from the Bothnian Bay and Bothnian Sea, where primary production is mostly P-limited (Tamminen and Andersen, 2007). However, nutrient limitation can switch to phosphorus during spring and autumn, in vicinity to freshwater inflows, and during blooms of Cyanobacteria, thus recommending the management measures for both nutrients (HELCOM, 2013).

In the Black Sea, both N and P play a role in the eutrophication processes (Black Sea Commission, 2008) so, consequently, both nutrients are used for classification by Romania and Bulgaria.

In the Mediterranean region, P is often the limiting nutrient, especially in the Eastern part (Thingstad et al., 2005), although both N and P are often co-limiting nutrients in this sea region (Dafner et al., 2003; Lazzari et al., 2016). Consequently, most countries use both N and P parameters, although Italy measures only P parameters in her coastal waters.

In the North East Atlantic region, N is generally assumed to be the limiting nutrient (Carstensen et al., 2011). Some North East Atlantic countries do not use P parameters for setting nutrient thresholds for coastal (five countries) and transitional waters (three countries). However, recent studies show that P may limit primary production (Karl, 2000), particularly in the vicinity of river plumes (Guillaud et al., 2008). TP was a better predictor for annual chl-a concentrations than TN in the Wadden sea, and N:P ratios in Danish coastal waters indicated combined N and P limitation with average TN:TP ratio (by mass) of 26.9 (Carstensen et al., 2011). Furthermore, the limiting nutrient can change both seasonally and spatially within this region (Burson et al., 2016).

In summary, an increasing body of evidence suggests that there may be drawbacks in relying upon a single limiting nutrient to achieve good ecological status, since limitation can vary both spatially and temporally in all water categories. However, as our study shows, assessment of N is neglected by many countries for inland waters, and P by several countries for coastal-transitional waters.

### 4.2 Different parameters and metrics used for nutrient criteria

#### 4.2.1 Lakes and rivers

Our results show that TP is the most widely-used P parameter for lakes, mostly measured as annual or growth season mean. There is less consistency in rivers, with countries using TP, TRP or SRP, either annual or growth season mean or 90th percentile values.

Similarly, TN is widely used in lakes while, for rivers, again, there is less consistency, with countries assessing only nitrate, only TN, or both forms together.

Science, tradition and pragmatism all play a part in the choice of parameters e.g., to ensure the continuity of long-term water quality datasets, which is important for detecting trends. In lakes, the total nutrient fraction is generally used, as long water retention times typically result in only a very small proportion of P being in the soluble form; the majority being incorporated in planktonic algal cells. In addition, the potential for rapid recycling of this biological nutrient fraction (Lyche et al., 1996) has meant that TP concentrations are a good reflection of P load in lakes and, thus, status assessment (OECD, 1982).

In many rivers, planktonic algae are less significant, the exception being large rivers where water retention time is sufficient to allow plankton communities to develop. In rivers, dissolved inorganic fractions of P and N represent a readily bioavailable fraction, in contrast to particulate and organic fractions, and are often a practical measure of nutrient pressure. This can be especially important in agriculturally-loaded rivers where stable particulate P, which is unavailable to support algal growth, may be the dominant fraction (Baker et al., 2014; Charles et al., 2019). There is, however, growing evidence showing that nutrients bound in organic complexes (“dissolved organic phosphorus”) can act as important resources for aquatic organisms (Burkholder et al., 2008; Whitton and Neal, 2011; Flynn et al., 2018).

While dissolved nutrient fractions have long been used for river monitoring and ensure continuity with historical records, there are situations where TP and TN may provide a more robust measure of nutrient supply. This might be case, for example, if low inorganic nutrient concentrations reflected high uptake and turnover rates in a water body that was, in fact, very productive (Dodds, 2003).

#### 4.2.2 Coastal and transitional waters

Our study shows many differences in the nutrient criteria used by countries in the assessment of saline waters, both concerning the assessment period (summer, winter or all year round), parameters (TN, TP, DIN, nitrate, SRP, nitrite, ammonium) and the statistical metrics (mean, median or 90th percentile). Not only are there differences between the countries, but there are also differences within the four marine ecoregions and even within countries between transitional and coastal waters.

Key eutrophication assessment parameters are winter DIN and SRP concentrations (recommended by HELCOM, 2015, HELCOM, 2017, and OSPAR, 2013). During phytoplankton blooms, dissolved inorganic nutrients in surface layers may be almost completely consumed, leading to large seasonal variability in nutrient concentrations (Nausch and Nausch, 2006). For this reason, Claussen et al. (2009) recommended that DIN and SRP should be assessed during winter, when biological activity is lowest. There are, however, exceptions: monitoring winter nutrient concentrations is not good practice for the western coastal areas of the Black Sea, as nutrient concentrations here peak in April–May at the time of highest Danube discharge (Black Sea Commission, 2008). Our review shows that inorganic nutrients are used by most countries (SRP by 14, DIN by 10 and nitrate by eight countries); however, only a few of them measure winter concentrations, while others measure annual or summer concentrations.

In the last decade, TN and TP, which include dissolved, particulate, inorganic and organic P and N fractions, have been increasingly used in coastal assessment, especially in the Baltic Sea (HELCOM, 2013). They are considered to be more robust parameters, less affected by seasonal nutrient conversion processes (Claussen et al., 2009). In addition, total nutrients are essential for determining nutrient budgets and establishing nutrient reduction targets. Our results show that total nutrients are assessed by many countries, but while some measure annual values (HELCOM, 2017), others measure summer or winter concentrations (Fig. 2, Table S2).

### 4.3 Comparison of nutrient thresholds within common types

There are a number of factors that complicate direct comparisons of nutrient thresholds between countries: different water body types, different summary statistics, different analytical techniques and parameters, and different approaches to establishing and using threshold concentrations. We have taken a pragmatic approach to the data, comparing threshold concentrations within broad types (Fig. 3, Fig. 4, Fig. 5) and with literature data linking nutrient concentrations to good ecological status (Table 4, Table 5, Table 6).

**Table 4:** Good-moderate class nutrient threshold values for lakes reported by member states compared with the values from the studies linking nutrient concentrations to good ecological status. MS – member states, TP - total phosphorus, TN – total nitrogen.

Lake broad type	Total phosphorus TP (μg L^−1^)		Total nitrogen TN (mg L^−1^)		Reference
	MS threshold values (range and [median])	Literature data	MS threshold values (range and [median])	Literature data	
2	9–140 [20]	18–20	0.4–4.0 [0.48]	0.7	Phillips et al., 2018
3	20–300 [44]	40	0.5–4.0 [1.6]	0.8–1.1	Phillips et al., 2018
		51		1.1–1.2	Poikane et al., 2019
		21–34		0.3–0.5	Dolman et al., 2016
4	20–300 [44]	52	0.5–4.0 [1.6]	1.1–1.5	Phillips et al., 2018
		58		1.0–1.4	Poikane et al., 2019
		41–74		0.7–1.1	Dolman et al., 2016
5	16–300 [27]	22–27	0.7–1.5 [0.7]	0.5–0.9	Phillips et al., 2018
8	11–70 [22]	14–32	–	–	Phillips et al., 2018
9	13–24 [18]	25	0.6	0.4–0.7	Phillips et al., 2018
13, 14	15–70 [29]	40	–	–	Marchetto et al., 2009

**Table 5:** Good-moderate class threshold values for rivers reported by member states compared with the values from the studies linking nutrient concentrations to good ecological status. MS – member states, SRP - soluble reactive phosphorus, TP - total phosphorus, TN – total nitrogen.

River broad type	Phosphorus (μg L^−1^)		Nitrogen (mg L^−1^)		Reference
	MS threshold values (range and [median])	Literature data	MS threshold values (range and [median])	Literature data	
1	SRP 70–310 [91]	46	NO_3_ 1.0–5.7 [2.0]	1.4	Phillips et al., 2018
1	TP 35–400 [150]	75	TN 0.7–10 [2.8]	–	Phillips et al., 2018
3	SRP 70–400 [82]	28–45	TN 0.5–10 [2.3]	1.1–3.5	Phillips et al., 2018
9	SRP 10–400 [82]	25–51	TN 0.4–10 [1.5]	1.7–2.5	Phillips et al., 2018

**Table 6:** Good-moderate class nutrient criteria for coastal and transitional waters reported by member states compared with the values from the literature (SRP – soluble reactive phosphorus; CW – coastal waters, TRW – transitional waters).

Common type	Total phosphorus (μg L^−1^)		Dissolved inorganic nitrogen (mg L^−1^)		Reference
	MS nutrient criteria	Literature data	MS nutrient criteria	Literature data	
CW BC1	13–19	7.4	0.10–0.18	0.039	HELCOM, 2015, HELCOM, 2017
CW BC4	15.5	21.7	0.15	0.073	HELCOM, 2015, HELCOM, 2017
CW BC5	27–33	23–25	0.11–0.15	0.036	Salas Herrero et al., 2019 (TP); HELCOM, 2015 (DIN)
CW BC7	SRP 15–24	SRP 9.3	0.10–0.23	0.035	HELCOM, 2015
CW BC9	16–22.3	17.1	0.18	0.037	HELCOM, 2015, HELCOM, 2017
CW MED I A	11.5–18.6	18			Salas Herrero et al., 2019
CW MED II A	13–18.6	13			Salas Herrero et al., 2019
TRW BT1	89–105	26–120	TN 0.3–1.1	TN 1.0–1.2	Salas Herrero et al., 2019

For lakes and rivers, the comparison was made possible by the following factors:

• Comparable parameters (TP and TN, mean or median, annual or growth season) are used by most countries;
• The European broad typology of 15 lake types and 20 river types encompasses most national types (Lyche Solheim et al., 2015, Lyche Solheim et al., 2019);
• Several studies have been carried out demonstrating the nutrient concentrations that support good ecological status (Dolman et al., 2016; Free et al., 2016: Phillips et al., 2018; Poikane et al., 2019).

#### 4.3.1 Lakes

Comparison of threshold concentrations in lakes shows that differences are partly a result of different lake types: siliceous and upland lakes have lower criteria than lowland and calcareous/mixed or organic lake types, reflecting well-established differences in background P loadings to these lake types (Cardoso et al., 2007; Poikāne et al., 2010).

However, there was a wide range of threshold values within lowland calcareous lake types (types 3, 4 and 6; Fig. 3). This is either a reflection of the wider range of conditions in what is, by definition, a “broad” typology; or of the use of different methods to derive the thresholds.

Most countries fall within the range defined by the studies linking nutrient concentrations to good ecological status (Table 4); however, some countries set threshold concentrations up to 300 μg TP L^−1^ and 4 mg TN L^−1^. These values are much higher than is suggested by the literature. For example, phosphorus levels supporting good ecological status for Irish lakes range from 16 to 30 μg TP L^−1^ (Free et al., 2016; different quality elements), for German lakes from 21 to 74 μg TP L^−1^ (Dolman et al., 2016; phytoplankton metrics in different lake types) and for UK lakes 11–66 μg TP L^−1^ (Willby et al., 2012; macrophytes in different lake types). In addition, several authors have shown that a linear relationship between nutrient concentrations and chlorophyll-a and phytoplankton species composition exists only up to 100 μg TP L^−1^ and 1.7 mg TN L^−1^ (Phillips et al., 2008), suggesting that other factors are likely to influence the productivity of lakes at higher concentrations.

#### 4.3.2 Rivers

There was much less evidence of type-specific differences for threshold concentrations in rivers and, for the majority of the types, the range of boundaries within the type were relatively high, up to 660 μg TP L^−1^ and 35 mg TN L^−1^ (both values 90th percentiles). There is much less literature available for river nutrient criteria than for lakes, linked to numerous difficulties developing pressure-response relationships (Dodds et al., 2002; Bowes et al., 2012). However, the threshold values supporting good ecological status provided by Phillips et al. (2018) are up to a magnitude lower than the highest of those proposed by some countries (Table 5).

It is important to recognize that rivers are highly heterogeneous systems which respond to a wide variety of pressures and local (physical) drivers which regulate primary production (Dodds and Welch, 2000; Bowes et al., 2016). Moreover, given the added complexity of a wider range of national-scale approaches to setting thresholds, a high variability in nutrient thresholds for rivers is not surprising. However, it is also possible that these results reveal a less well-developed view of the impact of nutrients in rivers than in lakes, and that further work to explore pressure-response relationships and interactions with other stressors, for a variety of biological quality elements is needed before realistic ranges of phosphorus thresholds can be established for all European rivers.

#### 4.3.3 Coastal and transitional waters

For coastal and transitional waters, comparison of nutrient criteria was severely hampered by several factors:

• differences in the nutrient parameters assessed by countries (only N or only P, different parameters, assessment seasons and metrics used);
• lack of broad types, leading to the use of common regional sea types, which are mostly shared by only a few countries (which, in turn, may not share nutrient parameters).

Regional Sea Conventions have provided guidelines in an attempt to harmonize assessment among their members (HELCOM, 2013; OSPAR, 2013); however, these have been followed only partially. For these reasons, comparisons of threshold concentrations have been limited. For instance, Latvia shares coastal water types with Estonia (BC4) and Lithuania (BC5) but a comparison has not been possible, as Latvia assesses winter DIN and SRP, while Lithuania and Estonia use annual TP and TN but do not share a common type with each other.

HELCOM has provided also good environmental status thresholds for open sea sub-regions (HELCOM, 2015, HELCOM, 2017) but our comparison reveals a diverse picture: most DIN criteria for coastal waters exceeds HELCOM thresholds by a factor 2–4, while TP criteria are mostly compliant or even stricter (BC4 type).

Very few studies define threshold concentrations supporting good ecological status in coastal waters. Of those that have been performed, Aigars et al. (2008) found that marked changes in biological communities occur at winter SRP concentrations >23 μg-P L^−1^ or 28 μg-P L^−1^ (southern and central part of the Gulf of Riga); these values correspond to the good-moderate threshold for regional type BC4 (23 μg L^−1^). Salas Herrero et al. (2019) derived good-moderate thresholds of 18 μg TP L^−1^ for MED Type IA and 13 μg TP L^−1^ for MED Type II A, which are similar to the TP criteria set by countries sharing these types (Table 6).

Deriving nutrient criteria for transitional waters (estuaries, coastal lagoons) presents significant problems that have not yet been fully solved (Reyjol et al., 2014). Our results show that high and very variable nutrient thresholds have been set for these complex and impacted ecosystems where the main difficulty is to distinguish between natural and anthropogenic stress (Elliott and Quintino, 2007). This is one of the knowledge gaps that must be addressed in the near future.

### 4.4 Different approaches to setting nutrient criteria

It is generally recognized that setting of nutrient thresholds should take into account biological responses to nutrient enrichment (Carvalho et al., 2013; Dolman et al., 2016; Poikane et al., 2019). However, in many cases we found that other approaches had been used. This was due to (i) lack of sufficient data from which empirical models of biological response to nutrient pressures for different water body types could be developed. (ii) Difficulties in establishing such models, especially for rivers (Dodds et al., 2002; Jarvie et al., 2013), coastal and transitional waters (Elliott and Quintino, 2007) and some lake types (Kelly et al., 2019). Consequently, countries used a wide variety of methods to establish threshold values that can be broadly divided in data-driven (regression, modelling, distribution of classified water bodies) and expert-judgement-based (as e.g. arbitrary divisions of distribution of nutrient concentrations in all water bodies).

For lakes, the most common approach is the use of regression models where nutrient concentration is related to a nutrient-sensitive BQE or part of a BQE such as chlorophyll *a* concentration or cyanobacteria abundance (Carvalho et al., 2013; Dolman et al., 2016). This approach is less common for rivers, probably due to weaker relationships between river BQEs and nutrients (Dodds et al., 2002). The relationships between nutrient concentrations and biological metrics are not as tightly coupled for rivers, where attached/benthic algae make a major contribution to primary production, and where other factors such as hydrodynamics, grazing pressures, riparian shading, and other anthropogenic pressures (such as toxic chemical discharges, changes to hydromorphology) can also regulate primary production (Munn et al., 2018).

For coastal and transitional waters, the approach most widely used is the so-called OSPAR Comprehensive Procedure where a water body is considered as a ‘Eutrophication Problem Area’ if the actual status deviates 50% or more from reference conditions (OSPAR, 2013). However, no agreement has been achieved on what constitutes reference conditions: there has been a wide range of historic years used to base background concentrations upon (e.g. 1880, 1900, 1950s, 1960s), even within a region and between neighboring countries. While this might be partly due to data availability, it also appears that there are very different notions among countries on background water quality conditions.

It should also be noted that the choice of 50% is not based on any scientific considerations about ecological changes caused by nutrient enrichment (Andersen et al., 2006) so is a sort of expert judgement. Therefore, HELCOM has developed an approach to setting thresholds for eutrophication parameters based on break points in long-term time series of these parameters, but only for the open sea basins of the Baltic Sea (HELCOM, 2013).

While threshold concentrations in coastal waters should also be based on knowledge of measurable biological response (Devlin et al., 2007) progress has been limited (but see Aigars et al., 2008; Salas Herrero et al., 2019). A common approach in the North Sea and Baltic Sea is to model historic nutrient inputs using catchment models and extrapolating these to coastal waters using a modelling approach or mixing diagrams (Schernewski et al., 2015; Ibisch et al., 2016; Blauw et al., 2019). This approach has been used to derive consistent boundaries for nutrients and chlorophyll-a, but has not included macrophytes and macrozoobenthos. This particular approach has been chosen since there is a general lack of current near-pristine conditions in coastal and marine waters as well as a lack of historic data on biological quality elements that go far enough back to represent near-pristine conditions.

In general, for both rivers and lakes, lower values (more stringent) were reported where data-driven (modelling or regression) methods were used to establish criteria values. The highest (i.e., more relaxed) thresholds were reported when statistical distributions and expert-based methods were used. This corresponds to previous findings that expert-based nutrient criteria must be used with caution as they may lead to less stringent threshold concentrations and a consequent failure to protect water quality (Dodds et al., 2006; Suplee et al., 2007).

For nitrate, the commonly-used values 5.65 and 11.3 mg NO_3_-N L^−1^ are derived from the guideline values of 25 and 50 mg-NO_3_ L^−1^ in the Drinking Water Directive (80/778/EC) and the Nitrates Directive (91/676/EC) respectively. However, the standards used for protection of drinking water supplies were not established with the objective of protecting good ecological status.

Given the uncertainty of nutrient/biological relationships and errors associated with determining ecological status, the task of setting threshold concentrations is clearly difficult. However, it is important that:

• the best available information and knowledge are used to derive nutrient criteria;
• the BQEs used to derive nutrient criteria are sensitive to nutrients;
• the most appropriate statistical techniques are used; if regression is not feasible, categorical methods can be used (Phillips et al., 2018);
• the resulting thresholds are broadly aligned with the wider body of published literature on nutrient limitation of primary production in different types of water bodies.

### 4.5 Implications for achieving ‘good’ ecological status

There are several implications that arise from setting inappropriate nutrient thresholds

1. Where threshold concentrations are too high (=relaxed), the criterion may be achieved without any biological response being observed. In this case, water bodies consequently fail to achieve good ecological status based on the BQE, even though they may meet the prescribed nutrient criterion.
2. Where nutrient criteria are based on a single element that is not the limiting nutrient (or which fails to account for P and N co-limitation); or on a nutrient fraction which is only a minor component of the total bioavailable nutrient pool and/or a poor indicator of overall nutrient supply (see above), there may again be no observed biological response. The Redfield ratio (Tett et al., 1985) offers a rapid check of the likelihood of N or P limitation that has practical benefits for determining appropriate mitigation measures (e.g. Burson et al., 2016).
3. Where nutrient thresholds are too low (i.e. too stringent), a water body might be classified as moderate status, despite the BQE corresponding to good ecological status. In such cases, the mismatch may cause measures to be implemented that are not strictly necessary.

There may also be other reasons why good ecological status has not yet been achieved, despite implementation of restoration measures, but which are not a result of inappropriate nutrient criteria; for example (see Jarvie et al., 2013):

• Inadequate intensity and targeting of restoration measures;
• Legacies of past land use management, which have accumulated nutrient stores within the catchment and can continue to impair water quality over timescales from years to decades and more (Carpenter, 2005; Sharpley et al., 2013)
• Decoupling of algal growth responses to nutrient loadings caused by a variety of factors, such as luxury uptake of phosphorus by algae during periods of high P availability, grazing pressure, physical controls such as flow regime, light availability and temperature, as well as other pressures such as toxic substances and hydromorphological alterations;
• Recovery trajectories which are non-linear and characterized by thresholds and alternative stable states.

## 5 Conclusions

### 5.1 Problems in a nutshell

The problems can be summarised as follows:

1. Different nutrients (N and/or P) are used for different water categories, based on prevailing assumptions that P is the most likely to be the limiting nutrient in freshwaters while N is likely to be limiting in coastal waters. Therefore, only P is used to set nutrient thresholds for many river and lake types, and only N for some coastal and transitional water types. Recent research has highlighted that co-limitation is more common than previously assumed so the use of a single nutrient criterion should always be questioned.
2. There are many differences in the nutrient parameters assessed (soluble or total), the assessment period (summer, winter or all year round), and in the metrics used (mean, median or 90th percentile), especially for coastal and transitional waters. These differences in nutrient criteria may hamper the comparison of threshold concentrations, definition of common objectives and a consistent management approach between countries and water categories.
3. In order to ensure good status is attained, nutrient criteria should be derived from biological responses to nutrients. However, a wide range of methods have been used by countries to set nutrient criteria, and their relationship to good ecological status is not always obvious. There is evidence that different approaches to criteria setting are likely to produce different results. For both rivers and lakes, the lowest threshold concentrations are associated with data-driven methods (modelling and regressions against nutrient sensitive biological quality elements), and the highest with expert judgement based methods (e.g., the use of the distribution of current nutrient concentrations measured across all water bodies in a country).
4. There are large variations in the nutrient threshold concentrations used to support good ecological status within shared types, especially for rivers and some types of lakes and transitional waters. Some variation is expected due to natural variation within broad types; however, we identify three major challenges for developing consistent and effective nutrient criteria across European countries: (i) Many countries report a single nutrient threshold concentration to protect good status for all their river types. (ii) Some countries use threshold concentrations that are significantly above known limiting nutrient concentrations (e.g. TP > 100 μg L^−1^ and TN > 1.7 mg L^−1^), reaching as high as 660 μg TP L^−1^ and 35 mg TN L^−1^; (iii) countries using N criteria taken from drinking water standards, which were not intended to support good ecological status.

Taken together, these findings provide evidence that not all national approaches to setting nutrient thresholds to support good ecological status are likely to achieve their goal.

### 5.2 Research agenda

#### 5.2.1 Establishing threshold concentrations for both P and N

Ample evidence has now accumulated to show that both P and N are capable of contributing to eutrophication and, therefore, that both need to be managed.

#### 5.2.2 Consistency in setting nutrient criteria

Consistent management of transboundary water bodies requires that nutrient parameters are monitored and assessed in a consistent manner between countries.

#### 5.2.3 Establishing the nutrient concentration that would support good ecological status

Threshold concentrations should be based on causal relationships between nutrient(s) and nutrient sensitive biological variables, taking into account the uncertainty of relationships and thus allowing a range of potential thresholds to be derived for specific water body types and circumstances (Phillips et al., 2018).

## Declaration of Competing Interest

The authors declare that they have no known competing financial interests or personal relationships that could have appeared to influence the work reported in this paper.

## Appendix A Supplementary data

Supplementary tables Image 1
