# Supplementary material for: Nutrient criteria for surface waters under the European Water Framework Directive: Current state-of-the-art, challenges and future outlook
Source: Sci Total Environ. 2019 Dec 10;695:133888. doi: 10.1016/j.scitotenv.2019.133888 (PMC6878824; doi:10.1016/j.scitotenv.2019.133888)
Supplement: Supplementary file 1 — Supplementary tables [file mmc1.docx]

Supplementary material for

**Nutrient criteria for surface waters under the European Water Framework Directive: Current state-of-the-art, challenges and future outlook**

Sandra Poikane*, Martyn G. Kelly, Fuensanta Salas Herrero, Jo-Anne Pitt, Helen P. Jarvie, Ulrich Claussen, Wera Leujak, Anne Lyche Solheim, Heliana Teixeira, Geoff Phillips

*Correspondence to: [sandra.poikane@ec.europa.eu](mailto:sandra.poikane@ec.europa.eu)

Table S1. Parameters and statistical metrics used to specify lake and river nutrient (phosphorus and nitrogen) boundary values for ecological classification under the European Water Framework Directive

TP - total phosphorus, SRP - soluble reactive phosphorus, TRP - total reactive phosphorus, TN - total nitrogen, NO_3_ - nitrates, Veg - vegetation season

|  | **Lakes** | | **Rivers** | |
| --- | --- | --- | --- | --- |
|  | **Phosphorus** | **Nitrogen** | **Phosphorus** | **Nitrogen** |
| Austria | Annual mean TP | Not used | Annual 90^th^ SRP | Annual 90^th^ NO_3_ |
| Belgium Flanders | Veg season mean TP | Veg season mean TN | Annual mean SRP  Veg season mean TP | Annual 90^th^ NO_3_  Veg season mean TN |
| Belgium Wallonia | No lakes | No lakes | Annual 90^th^ TP | Annual 90^th^ NO_3_ |
| Bulgaria | Annual mean TP  Annual mean SRP | Annual mean TN  Annual mean NO_3_ | Annual mean TP  Annual mean SRP | Annual mean TN  Annual mean NO_3_ |
| Cyprus | Annual mean TP | Not used | Annual mean TP  Annual mean SRP | Annual mean NO_3_ |
| Czech Republic | Veg season mean TP | Not used | Annual median TP  Annual median SRP | Annual max NO_3_  Annual median NO_3_ |
| Germany | Veg season median and 75^th^ percentile TP | Not used | Annual mean TP  Annual mean SRP | Not used |
| Denmark | Veg season mean TP | Veg season mean TN | Not used | Not used |
| Estonia | Veg season mean TP | Veg season mean TN | Annual mean TP | Annual mean TN |
| Spain | Annual 75th  percentile TP | Not used | Annual mean SRP | Annual mean NO_3_ |
| Finland | Veg season mean TP | Veg season mean TN | Annual mean TP | Annual mean TN |
| France | Veg season median TP | Veg season max NO3 | Annual 90^th^ SRP  Annual 90^th^ TP | Annual 90^th^ NO_3_ |
| Greece | Annual mean TP | Annual mean TN | Annual mean TP | Annual mean NO_3_ |
| Croatia | Veg season mean TP | Veg season mean NO_3_ | Annual median TP | Annual median TN  Annual median NO_3_ |
| Hungary | Annual mean TP  Annual mean SRP | Annual mean TN  Annual mean NO_3_ | Annual mean TP  Annual mean SRP | Annual mean TN  Annual mean NO_3_ |
| Ireland | Annual mean TP | Not used | Annual 95^th^ TRP  Annual mean TRP | Annual mean NO_3_ |
| Italy | Annual mean TP | Not used | Annual mean TP | Annual mean NO_3_ |
| Lithuania | Veg season mean TP | Veg season mean TN | Annual mean TP  Annual mean SRP | Annual mean TN  Annual mean NO_3_ |
| Luxemburg | No lakes | No lakes | Annual mean TP  Annual mean SRP | Annual mean NO_3_ |
| Latvia | Annual mean TP | Annual mean TN | Annual mean TP | Annual mean TN |
| Malta | Not reported | Not reported | Not reported | Not reported |
| Netherlands | Veg season mean TP | Veg season mean TN | Veg season mean TP | Annual men TN |
| Norway | Veg season mean TP | Veg season mean TN | Annual mean TP | Annual mean TN |
| Poland | Veg season mean TP | Veg season mean TN | Veg season mean TP  Veg season mean SRP | Veg season mean TN  Veg season mean NO_3_ |
| Portugal | Annual mean TP | Annual mean TN  Annual mean NO_3_ | Annual mean TP | Annual mean NO_3_ |
| Romania | Veg season mean TP  Veg season mean SRP | Veg season mean TN  Veg season mean NO_3_ | Veg season mean TP  Veg season mean SRP | Annual 90^th^ NO_3_  Annual 90^th^ TN |
| Sweden | Veg season mean TP | Not used | Annual mean TP | Not used |
| Slovenia | Veg season mean TP | Not used | Annual median TP | Annual median NO_3_ |
| Slovakia | No lakes | No lakes | Annual 90th TP  Annual 90th SRP | Annual 90^th^ NO_3_  Annual 90^th^ TN |
| United Kingdom | Annual mean TP | Not used | Annual mean TRP | Not used |

Table S2. Metrics used to specify coastal and transitional waters nutrient (phosphorus and nitrogen) boundary values for ecological classification under the European Water Framework Directive

TP - total phosphorus, SRP - soluble reactive phosphorus, TN - total nitrogen, DIN – dissolved inorganic nitrogen, KjN – Kjeldahl nitrogen, NO_3_ - nitrates, TRW – transitional waters, MED – Mediterranean Sea region, NEA – North East Atlantic Sea region

|  | **Coastal waters** | | **Transitional waters** | | | |
| --- | --- | --- | --- | --- | --- | --- |
|  | **Phosphorus** | **Nitrogen** | **Phosphorus** | | **Nitrogen** | |
| Belgium Flanders | Winter mean SRP | Winter mean TN | Annual mean SRP  Veg season mean TP | | Annual 90th NO_3_  Winter mean DIN  Veg season mean TN  Annual 90th KjN | |
| Bulgaria | Spring, summer, autumn, winter max SRP | Spring, summer, autumn, winter max NO_3_ | Annual mean SRP  Annual mean TP | | Annual mean NO_3_  Annual mean TN | |
| Cyprus | Annual mean SRP | Annual mean NO_3_ | TRW not defined | | TRW not defined | |
| Germany (Baltic) | Annual mean TP | Annual mean TN | TRW not defined | | TRW not defined | |
| Germany (NEA) | Annual mean TP  Winter mean SRP | Winter mean DIN  Annual mean and veg season TN | Annual mean TP Winter mean SRP | | Winter mean DIN  Annual mean TN | |
| Denmark | Do not set limits but loads | Do not set limits but loads | TRW not defined | | TRW not defined | |
| Estonia | Summer mean TP | Winter mean DIN  Summer mean TN | TRW not defined | | TRW not defined | |
| Spain (NEA) | Annual mean SRP | Annual mean NO_3_ | Annual mean SRP | | Annual mean NO_3_ | |
| Spain (MED) | Annual mean SRP | Annual mean NO_3_ | Annual mean TP | | Annual mean TN | |
| Finland | Summer mean TP | Summer mean TN | TRW not defined | | TRW not defined | |
| France (MED) | Not derived yet | Not derived yet | Summer 90^th^ TP  Summer 90^th^ SRP | | Summer 90^th^ TN  Summer 90_th_ DIN | |
| France (NEA) | Not used | Winter mean DIN | not used | | Winter mean DIN | |
| Greece | Annual mean SRP | Annual mean NO_3_ | Annual mean SRP | | Annual mean NO_3_ | |
| Croatia | Annual median SRP  Annual median TP | Annual median DIN | Annual median SRP Annual median TP | | Annual median DIN | |
| Ireland | Not used | Summer median DIN Winter median DIN | Summer median SRP  Winter median SRP | | Not used | |
| Italy | Annual mean TP | Not used | Annual median SRP | | Annual mean DIN | |
| Lithuania | Summer mean TP | Summer mean TN | Summer mean TP | | Summer mean TN | |
| Latvia | Winter mean SRP | Winter mean DIN | Winter mean SRP | | Winter mean DIN | |
| Netherlands | Not used | Winter mean DIN | Not used | | Winter mean DIN | |
| Norway | Summer and winter  mean SRP  Summer and winter  mean TP | Summer and winter  mean NO_3_  Summer and winter  mean TN | TRW not defined | | TRW not defined | |
| Poland | Summer mean TP  Winter mean SRP | Summer mean TN  Winter mean DIN  Winter mean NO_3_ | | Annual or summer mean TP  Annual or winter mean SRP | | Annual or summer  mean TN  Annual or winter mean  DIN and NO_3_ |
| Portugal | Annual 90^th^ SRP | Annual 90th NO_3_ Summer mean TN  Winter mean TN | Annual 90^th^ SRP | | Annual 90^th^ NO_3_ | |
| Romania | TP | NO_3_ | TP | | NO_3_ | |
| Sweden | Winter mean SRP Summer mean TP  Winter mean TP | Winter mean DIN Summer mean TN  Winter mean TN | Winter mean SRP Summer mean TP Winter mean TP | | Winter mean DIN  Summer mean TN  Winter mean TN | |
| Slovenia | Annual mean TP  Annual mean SRP | Annual mean NO_3_ | TRW not defined | | TRW not defined | |
| United Kingdom | Not used | Winter 99^th^ DIN  Winter mean DIN | Not used | | Winter 99^th^ DIN  Winter mean DIN | |

Table S3. Description of broad types for lakes with number of countries and national lake types with reported nutrient thresholds, allocated to each broad type.

Geology: calcareous – alkalinity > 1 mEq/L; siliceous – alkalinity < 1 mEq/L; organic – colour > 30 mgPt/L

|  |  | Altitude  (m asl) | Lake area  (km^2^) | Mean depth  (m) | Total phosphorus | | Total nitrogen | |
| --- | --- | --- | --- | --- | --- | --- | --- | --- |
| Type code | Broad Type |  |  |  | Countries | National types | Countries | National types |
| 1 | Very large and deep (stratified) (all Europe) | any | > 100 | >3 | 4 | 5 | 2 | 3 |
| 2 | Lowland, siliceous | <200 | < 100 | >3 | 7 | 32 | 4 | 9 |
| 3 | Lowland, stratified, calcareous/mixed | <200 | < 100 | >3 | 16 | 41 | 11 | 33 |
| 4 | Lowland, calcareous/mixed, very shallow (unstratified) | <200 | < 100 | ≤ 3 | 14 | 33 | 11 | 23 |
| 5 | Lowland, organic and siliceous | <200 | < 100 | >3 | 5 | 25 | 4 | 8 |
| 6 | Lowland, organic and calcareous/mixed | <200 | < 100 | >3 | 6 | 13 | 4 | 6 |
| 7 | Mid altitude, siliceous | 200-800 | < 100 | >3 | 9 | 21 | 4 | 13 |
| 8 | Mid altitude, calcareous-mixed | 200-800 | < 100 | >3 | 8 | 26 | 3 | 14 |
| 9 | Mid altitude, organic-siliceous | 200-800 | < 100 | >3 | 22 | 8 | 1 | 2 |
| 10 | Mid altitude, organic & calcareous/mixed | 200-800 | < 100 | >3 | 1 | 1 | - | - |
| 11 | Highland, siliceous (all Europe) | >800 | < 100 | >3 | 6 | 15 | 4 | 16 |
| 12 | Highland, calcareous/mixed (all Europe) | >800 | < 100 | >3 | 2 | 2 | - | - |
| 13 | Mediterranean, small-large, siliceous (including reservoirs) | <800 | 0.5-100 | any | 3 | 6 | 2 | 4 |
| 14 | Mediterranean, small-large, calcareous-mixed (including reservoirs) | <800 | 0.5-100 | any | 4 | 11 | 2 | 8 |
| 15 | Mediterranean, very small | <800 | <0.5 | <15 | 1 | 1 | 1 | 1 |

Table S4. Description of broad types for rivers with number of countries and national river types with reported nutrient thresholds, allocated to each broad type.

Geology: calcareous – alkalinity > 1 mEq/L; siliceous – alkalinity < 1 mEq/L; organic – colour > 30 mgPt/L

| Type code | Broad Type | Altitude  (m asl) | Catchment area  (km^2^) | Total phosphorus | | Total nitrogen | |
| --- | --- | --- | --- | --- | --- | --- | --- |
|  |  |  |  | Countries | National types | Countries | National types |
| 1 | Very large rivers (all Europe) | any | >10 000 | 12 | 23 | 11 | 21 |
| 2 | Lowland, siliceous, medium-large | <200 | 100-10 000 | 6 | 16 | 4 | 10 |
| 3 | Lowland, siliceous, very small-small | <200 | <100 | 11 | 34 | 7 | 22 |
| 4 | Lowland, calcareous/mixed, medium-large | <200 | 100-10 000 | 19 | 95 | 16 | 80 |
| 5 | Lowland, calcareous/mixed, very small-small | <200 | <100 | 20 | 62 | 16 | 35 |
| 6 | Lowland, organic & siliceous | <200 | <10 000 | 6 | 19 | 3 | 6 |
| 7 | Lowland, organic & calcareous/mixed | <200 | <10 000 | 3 | 11 | 1 | 1 |
| 8 | Mid altitude, siliceous, medium-large | 200-800 | 100-10 000 | 9 | 19 | 6 | 13 |
| 9 | Mid altitude, siliceous, very small-small | 200-800 | <100 | 12 | 38 | 9 | 33 |
| 10 | Mid altitude, calcareous/mixed, medium-large | 200-800 | 100-10 000 | 12 | 47 | 10 | 39 |
| 11 | Mid altitude, calcareous/mixed, very small-small | 200-800 | <100 | 11 | 45 | 9 | 40 |
| 12 | Mid altitude, organic & siliceous | 200-800 | <10 000 | 3 | 7 | 2 | 3 |
| 13 | Mid altitude, organic & calcareous/mixed | 200-800 | <10 000 | 2 | 3 | 1 | 1 |
| 14 | Highland (all Europe), siliceous | >800 | <10 000 | 7 | 16 | 6 | 15 |
| 15 | Highland (all Europe), calcareous/mixed | >800 | <10 000 | 8 | 15 | 8 | 20 |
| 16 | Glacial rivers (all Europe) | >200 | <10 000 | 2 | 12 | 2 | 12 |

Table S5. Description of common types for coastal and transitional waters with number of countries and national types with reported nutrient thresholds, allocated to each common type.

| Regional Sea | Type code | Common type | Phosphorus (TP) | | Nitrogen (DIN) | |
| --- | --- | --- | --- | --- | --- | --- |
|  |  |  | Countries | National types | Countries | National types |
| Baltic | BC1 | Sites in the Quark and the Bothnian Sea, extending to the Archipelago Sea | 2 | 10 | 1 | 8 |
|  | BC3 | Finnish and Estonian coasts of Gulf of Finland | 2 | 3 | 1 | 2 |
|  | BC4 | Sites of Estonia and Latvia in the Gulf of Riga | 1 | 2 | 2 | 4 |
|  | BC5 | Sites in the southeastern Baltic Sea along the coast of Latvia, Lithuania and Poland | 2 | 4 | 2 | 4 |
|  | BC6 | Sites along the Western Baltic Sea at the southern Swedish coast and the southeastern Danish coast | 1 | 3 | 1 | 3 |
|  | BC7 | Western Polish coast and eastern German coast | 2 | 2 | 1 | 1 |
|  | BC8 | Danish and German coasts in the Western Baltic Sea | 1 | 3 | - | - |
| North East Atlantic | NEA1/26 | Open oceanic or enclosed seas, exposed or sheltered, euhaline, shallow | 2 | 8 | 4 | 102 |
|  | NEA3/4 | Polyhaline, Exposed or moderately exposed (Wadden Sea type) | 1 | 2 | 1 | 1 |
|  | NEA7 | Deep fjordic and sea loch systems | 1 | 3 | 1 | 1 |
|  | NEA8a | Skagerrak Inner Arc Type, polyhaline, microtidal, moderately exposed, shallow | 1 | 1 | 1 | 1 |
|  | NEA8b | Skagerrak Inner Arc Type, polyhaline, microtidal, moderately sheltered, shallow | 1 | 4 | 1 | 4 |
|  | NEA9 | Fjord with a shallow sill at the mouth with a very deep maximum depth in the central basin with poor deepwater exchange | 1 | 1 | 1 | 1 |
|  | NEA10 | Skagerrak Outer Arc Type, polyhaline, microtidal, exposed, deep | 1 | 1 | 1 | 1 |
| Mediter  ranean | Type I | Highly influenced by freshwater input | 1 | 4 | - | - |
|  | Type IIA, | Moderately influenced by freshwater input (continent influence) | 2 | 9 | 1 | 5 |
|  | Type IIIW | Continental coast, not influenced by freshwater input (Western Basin). | 1 | 4 | 1 | 24 |
|  | Type IIIE | Not influenced by freshwater input (Eastern Basin) |  | - | 1 | 1 |
| Black Sea | CW-BL1 | Mesohaline, microtidal (< 1 m), shallow (< 30 m), moderately exposed, mixed substratum | - | - | - | - |
|  |  | Not allocated |  | 47 |  | 18 |
|  |  | Total reported | 10 | 113 | 10 | 183 |

Table S6. Description of common types for transitional waters with number of countries and national types with reported nutrient thresholds, allocated to each common type.

| Regional Sea | Type code | Common type | Total phosphorus (TP) | | Dissolved inorganic nitrogen (DIN) | |
| --- | --- | --- | --- | --- | --- | --- |
|  |  |  | Countries | National types | Countries | National types |
| Baltic | BT1 | Polish Vistula lagoon and Lithuanian Curonian lagoon | 2 | 6 | 1 | 1 |
| North East Atlantic | NEA11 | Transitional Waters | 1 | 9 | 2 | 8 |
| Mediterranean Sea | MED Transitional waters | Coastal lagoons |  |  | 2 | 5 |
| Mediterranean Sea | MED Transitional waters | Estuaries | 1 | 2 | 1 | 4 |
|  |  | Not allocated |  | 14 |  | 10 |
|  |  | Total reported | 4 | 27 | 6 | 28 |
